# Morphological Adaptations for Shell Anchoring in *Calcinus tibicen*: Insights From µCT Imaging, Histology and Scanning Electron Microscopy

**DOI:** 10.1002/jmor.70112

**Published:** 2026-01-13

**Authors:** Alexandre R. da Silva, Carolina Siqueira Safra Terra Melo, Giselle Pinto de Faria Lopes, Caio S. Nogueira

**Affiliations:** ^1^ Museu de Zoologia da Universidade de São Paulo (MZUSP), Universidade de São Paulo (USP) São Paulo São Paulo Brazil; ^2^ Marine Biotechnology Department Instituto de Estudos do Mar Almirante Paulo Moreira, IEAPM Arraial do Cabo Rio de Janeiro Brazil; ^3^ Invertebrate Morphology Laboratory (IML), Department of Biology School of Agricultural and Veterinary Sciences (FCAV) São Paulo State University (UNESP) Jaboticabal São Paulo Brazil

**Keywords:** µCT, crustacea, hermit crabs, setae morphology, shell anchoring

## Abstract

Hermit crabs rely on external shells for protection due to their non‐calcified pleons. This study focuses on the anatomical features and functional roles of various appendages in *Calcinus tibicen* to understand their mechanisms for shell anchoring. Using scanning electron microscopy (SEM), histological analyses and micro‐computed tomography (µCT), we examined the morphology and internal structure of the fourth and fifth pereopods, telson, and uropods. SEM revealed that the pereopods are equipped with scale setae and teeth, which facilitate a firm grip on the shell's internal surface. µCT imaging showed that the uropods play a critical role in gripping the shell, with the left uropod exhibiting more developed musculature. Histological analysis showed that the muscles of the tailfan are striated and also revealed the presence of connective, hemolymphatic and, epithelial tissues. These findings enhance our understanding of the morphological adaptations that facilitate shell use in hermit crabs, emphasizing the importance of both external and internal structures in maintaining grip and stability. This study fills gaps in the literature regarding the role of the tailfan and pereopods in hermit crab shell anchoring, suggesting that uropods function as hooks, and the fourth and fifth pairs of pereopods act as supporting structures.

## 1 Introduction

Different from other decapod crustaceans, hermit crabs lack a fully calcified pleon (Hazlett 1966; 1981; Kellogg 1976; Tsang et al. 2011). As a result, these animals rely on external structures for pleon protection and shelter (Williams and McDermott 2004), being empty gastropod shells the most commonly used structures (Hazlett 1966; Kellogg 1976, 1977).

The availability of suitable shells is crucial for hermit crabs throughout their entire life cycle (Angel 2000; da Silva et al. 2018; Torjman and Iyengar 2022). These animals require shells with appropriate weight, size and internal volume for proper development (Osorno et al. 1998; Angel 2000; Sato et al. 2005; da Silva et al. 2018; Alcaraz and Kruesi 2019; Kruesi et al. 2022). Shell selection is not random, and factors such as the presence of epibionts, fissures, and weight are taken into consideration (Jackson and Elwood 1989; Buckley and Ebersole 1994; Carlon and Ebersole 1995; Osorno et al. 2005; Ragagnin et al. 2016; Stanski et al. 2018; Suárez‐Rodríguez et al. 2019; da Silva et al. 2022). The condition of the shell can directly affect hermit crab life, potentially limiting their growth and reproduction (Angel 2000; da Silva et al. 2018; Burciaga et al. 2021). However, the availability of well‐suited shells is not always guaranteed for the hermit crab community in a given area (Sato et al. 2005; da Silva et al. 2020, 2021; Kruesi et al. 2022; Torjman and Iyengar 2022).

The coexistence of hermit crabs within a particular habitat is frequently influenced by both inter‐ and intraspecific competition (Kellogg 1977; Sant'Anna et al. 2012; Kruesi et al. 2022; Torjman and Iyengar 2022; da Silva et al. 2023). The acquisition of shells can occur through a clustering behavior known as the “Shell exchange market” (Gherardi and Vannini 1993) or through agonistic interactions (Bertness 1981; Lane and Briffa 2020). In these interactions, hermit crabs often strike opponents with their shells, a behavior known as rapping (Lane and Briffa 2020; Lane et al. 2022). The goal of this hit is to make its opponent lose grip of the anchor point inside the shell, allowing the attacker to potentially acquire a new shell (Lane and Briffa 2020; Lane et al. 2022).

To ensure better adherence to the shell and to facilitate both movement over the substrate and participation in agonistic interactions, anchorage within the inner part of the shell is extremely important. The pleonal musculature surrounds the columella of the shell (Elmhirst 1947; Brightwell 1951; Chapple 1966a, 1969a; Lane and Briffa 2020), while the fourth and fifth pairs of pereiopods support the shell through a set of specialized setae (Elmhirst 1947; Bott 1949; Brightwell 1951). The importance of these appendages is so remarkable that morphological vestiges related to their supporting mechanism can still be observed in carcinized anomuran groups, such as lithodids (Richter and Scholtz 1994). Nevertheless, the exact role of the tailfan, composed of the telson and uropods, remains unclear. However, it has been suggested that the tailfan acts as a hook, enhancing the grip of the hermit crab inside the shell (Bott 1949; Brightwell 1951).

The mechanical and motor capabilities of hermit crabs' pleon in holding the shell have been extensively studied (Chapple 1966b, 1966a, 1969a, 1969b, 1973, 1993; Bent and Chapple 1977). The external morphology of hermit crabs' pleon and tailfan exhibits apparent asymmetry (Chapple 1966a, 1969b), and studies have shown that one side of the internal musculature possesses more developed muscles (Chapple 1966a, 1969b), which may be associated with the more developed uropod. However, the muscle pattern of the uropods remains unknown. The observation of hermit crabs' behavior using glass shells has provided further evidence of the pleon's importance in shell anchoring, but the role of the tailfan in this process remains poorly understood (Thorson 1946; Elmhirst 1947; Bott 1949; Brightwell 1951).

Therefore, the present study describes the external morphology of the fourth and fifth pairs of pereopods and the tailfan to identify structures that serve as hooks and anchor points. Also, the tailfan's internal morphology was analyzed via micro‐computed tomography (µCT) scan and histological analyses to understand how its internal musculature is organized and connected to the axial body.

## 2 Material and Methods

For the study, the species *Calcinus tibicen* (Herbst, 1791) was used as a model. This species has a wide distribution in the Atlantic Ocean, occurring along the coastal regions of the Western Atlantic from the United States to the southern region of Brazil (Mandai et al. 2018). Additionally, this organism can utilize a variety of shells across its distribution, and its biology is well‐known (Leite et al. 1998; Mantelatto and Garcia 1999; Garcia and Mantelatto 2001; Batista‐Leite et al. 2005; Marcondes et al. 2018).

The morphology of all structures that constitute the hermit crabs' anchoring mechanism within the shells was investigated, including the fourth and fifth pairs of pereopods (P4 and P5), the telson, and the uropods (Thorson 1946; Bott 1949; Brightwell 1951; Chapple 1973). Throughout the manuscript, we use the term tailfan to collectively refer to the uropods and telson, for the sake of simplicity in data presentation. However, it is important to clarify that the tailfan in hermit crabs does not represent a true tailfan, such as that observed in shrimp‐like crustaceans exhibiting the characteristic caridoid escape reaction (Heitler et al. 2000). Only the propodus and dactyl of both pereopods (P4 and P5) were analyzed, as they are the most distal articles directly in contact with the internal part of the shell, acting as an anchoring mechanism (Bott 1949; Brightwell 1951; Richter and Scholtz 1994; Lane et al. 2022). The morphology of these structures was analyzed through scanning electron microscopy (SEM) and µCT. The aim of these analyses was to investigate the morphological characteristics of these structures that may assist hermit crabs in anchoring themselves within their shells.

### 2.1 External Anchor Structures

SEM analyses were conducted to describe the morphological characteristics of external anchoring in each structure. These structures are referred to herein as the scales and setae present on the animals' exoskeleton and are potentially used for enhancing grip on shells. Thus, for this purpose, two specimens had their P4 and P5, as well as the tailfan (uropods + telson), carefully dissected from their bodies and subsequently cleaned with a brush. The appendages were then dehydrated through a series of ethanol washes (25%, 50%, 75%, and 100%) and critically dried with liquid CO2.

Subsequently, all structures (P4, P5, uropod, and telson) were individually mounted on aluminum stubs and analyzed using a LEO 440 scanning electron microscope. The specimens used are part of the Carcinology collection at the Zoology Museum of the University of São Paulo (MZUSP 43089 and MZUSP 43090). The description of the setae present in these structures followed the terminology proposed by Fleischer et al. (1992) and Keiler and Richter (2011).

### 2.2 Internal Morphology and Position of Uropods

Since the uropods are positioned at different planes (see results), a hermit crab was scanned inside the shell with µCT to evaluate the positioning of the uropods against the grain of the shells. A specimen of *C. tibicen* was sampled inside a Muricidae shell and was frozen in the lab to preserve its position inside the shell. Since the goal of this scan was not to evaluate internal structures, the specimen was not stained. The hermit crab inside the shell was placed inside a plastic tray and taken to µCT scan with micro focal (GE phoenix v|tome|x). The scanning was conducted with the following parameters: a detector size of 2014 × 2014 pixels, a voltage of 70 kV, a current of 170 µA, a voxel size of 3.2 µm, and a total of 1400 images were captured. Each image had an exposure time of 1000 ms. The 3D visualization and reconstruction were performed using the 3D Slicer software (Fedorov et al. 2012).

To assess the internal musculature arrangement of the uropod, a fresh specimen of *C. tibicen* was anesthetized using cold temperature and its pleon was carefully dissected from the body. The pleon was fixed in 70% ethanol and then stained with IKI (1% iodine metal (I2) + 2% potassium iodide (KI) in water) following the method described by Metscher (2009). Subsequently, the stained pleon was placed in a sealed plastic tray and scanned using a µCT system with a micro focal point (GE phoenix v|tome|x). The scanning was performed using the same parameters mentioned earlier.

### 2.3 Histological Characterization of the Tailfan Tissues

Since the tailfan structures are positioned at the pleon‐shell transition, histological procedures were performed to characterize the tissue types and to associate them with other morphological and functional results. Briefly, the samples were decalcified in a solution of 10% formic acid and 5% formaldehyde until the carapace was completely dissolved. The fixed material was then dehydrated using an increasing series of ethyl alcohols (60%, 70%, 80%, 90%, 100%, and 100%) for 40 min at each step. For the clearing process, samples were submerged in two series of xylene for 30 min each. Subsequently, the material was embedded in two baths of paraffin for 1 h each and finally cut into 6 µm sections. Slides were then stained with hematoxylin and eosin (Souza et al. 2017).

## 3 Results

### 3.1 External Anchor Structures (SEM)

**Figure 1:**
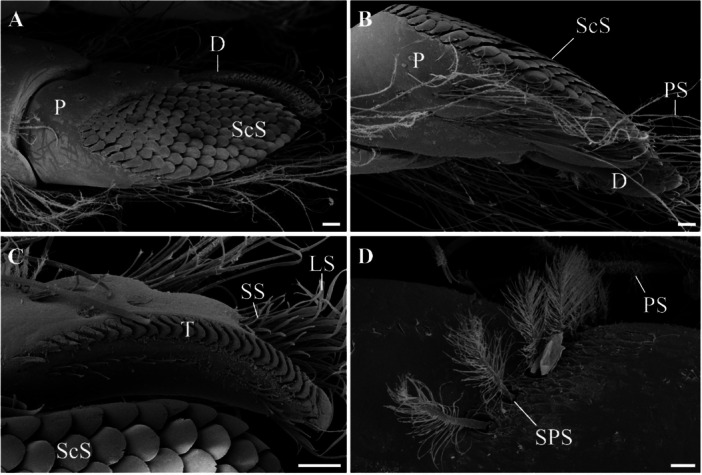
Fourth pereopod of *Calcinus tibicen* (Herbst, 1791). (A) General view of the propodus and dactyl, showing the presence of “rasping” structures, which are actually scale setae. (B) Lateral view of the propodus, dactyl, and scale setae. Additionally, pappose setae can be observed in this region. (C) Detail of the dactyl, displaying a row of overlapping multiple teeth. Furthermore, long and short simple setae associated with the medial and distal region of this article can be observed. (C) Dorsal view of the propodus, revealing the presence of plumose setae. The scale bars in A, B, and C correspond to 100 µm, and the scale bar in D corresponds to 20 µm. D, Dactyl; LS, Long simple seta; P, Propodus; PS, Papillate seta; ScS, Scale seta; SPS, Plumose seta; SS, Short simple seta; T, Teeth.

The fourth pair of pereopods exhibits numerous long setae arising from the midsection of the propodus (Figure 1A). In the dorsal view, distinct “rasping” structures mentioned by Bott (1949) can be observed. Upon close visualization, these “rasping” structures are identified as scales setae (ScS) (Figure 1A), which overlap with each other (Figure 1B). They are oriented against the shell's grains, potentially facilitating a better grip (Figure 1A,B). The dactyl of the pereopod displays multiple overlapping teeth also oriented against the shell's grain (Figure 1C). The propodus and dactyl of the fourth pair of pereopods exhibit short setae (SS) and long simple setae (LS), pappose setae (PS) and short plumose setae in dorsal view (SPS) (Figure 1C,D).

**Figure 2:**
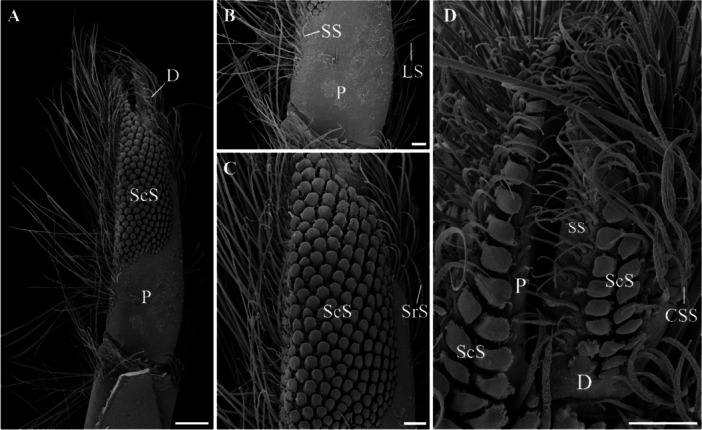
Fifth pereopod of *Calcinus tibicen* (Herbst, 1791). (A) General view of the propodus and dactyl. A wide variety of setae can be observed on these two articles, with particular emphasis on the presence of scale setae in both articles. (B) Detailed view of the proximal region of the propodus. (C) Detailed view of the distal region of the propodus. (D) Detailed view of the distal region of the propodus and dactyl. The scale bar in A corresponds to 300 µm, while the scale bars in B, C, and D correspond to 100 µm. CSS, Composite serrate seta; D, Dactyl; LS, Long simple seta; P, Propodus; ScS, Scale seta; SrS, Serrate seta; SS, Short simple seta.

The fifth pair of pereopods also presents a wide variety of setae from the proximal region of the propodus to the distal region of the dactyl (Figure 2A). In this appendage, ScS are observed in both the propodus and the dactyl (Figure 2A). Additionally, there are SS, LS, CSS, and SrS distributed along these two articles (Figure 2B–D).

**Figure 3:**
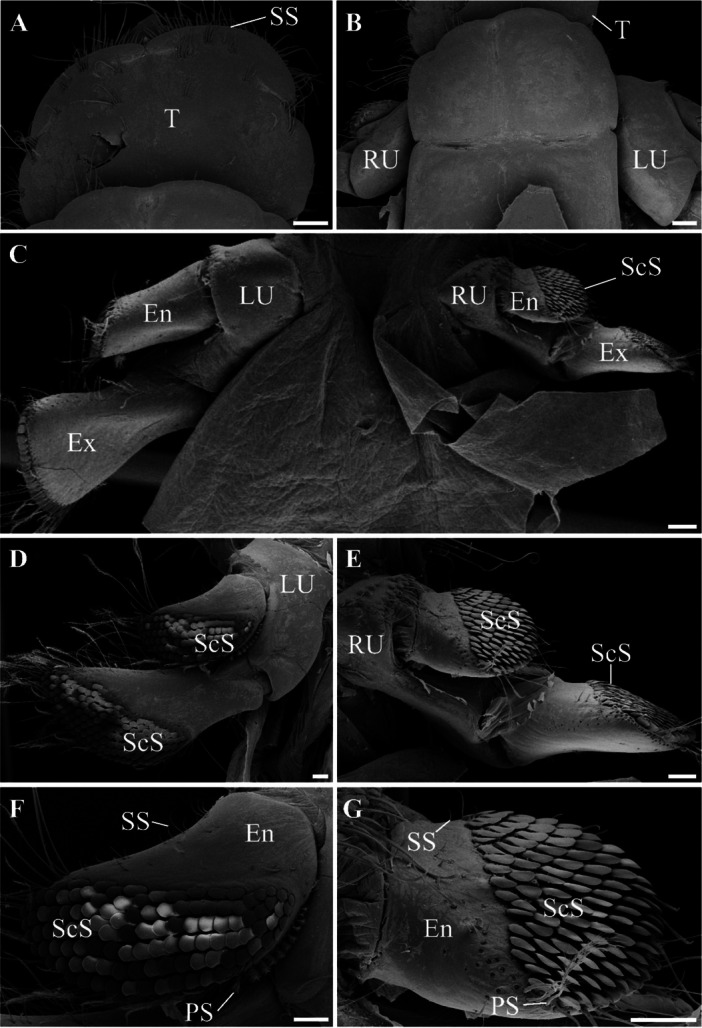
Tailfan of *Calcinus tibicen* (Herbst, 1791). (A) Detail of the telson. The presence of scattered short simple setae along the margin of this structure can be observed. (B) Anterior region to the telson, where the pair of uropods is articulated. (C) General ventral view of the uropods (left and right). The uropods consist of an endopod and exopod. The presence of scale setae can be noted on these structures. (D, E) Detail of the left and right uropods, respectively. The presence of scale setae on the endopods and exopods of both uropods can be observed. (F, G) Detail of the endopods of the left and right uropods, respectively. The presence of scale setae, plumose setae, and short simple setae can be noted. The scale bar in A, B, and C corresponds to 300 µm, while the scale bar in D, E, F, and G corresponds to 200 µm. En, Endopod; Ex, Exopod; LU, Left uropod; PS, Plumose setae; RU, Right uropod; ScS, Scale setae; SS, Short simple setae; T, Telson.

The telson exhibits a predominantly smooth surface, with scattered short simple setae along its margin (Figure 3A). Anterior to the telson, the pair of uropods can be observed (left and right; Figure 3B), together forming the tailfan (telson + uropods). There are differences in size between the uropods, with the left uropod being larger than the right uropod (Figure 3C). These structures consist of endopod and exopod, with the endopod being smaller compared to the exopod. Furthermore, in the case of hermit crabs, the endopods and exopods of both uropods are arranged in different planes (Figure 3C). ScS can be observed on the uropods, both on the endopod and exopod, regardless of the side (Figure 3D,E). Additionally, the presence of SS and SPS is also observed (Figure 3F,G).

### 3.2 Internal Morphology and Position of Uropods (µCT)

**Figure 4:**
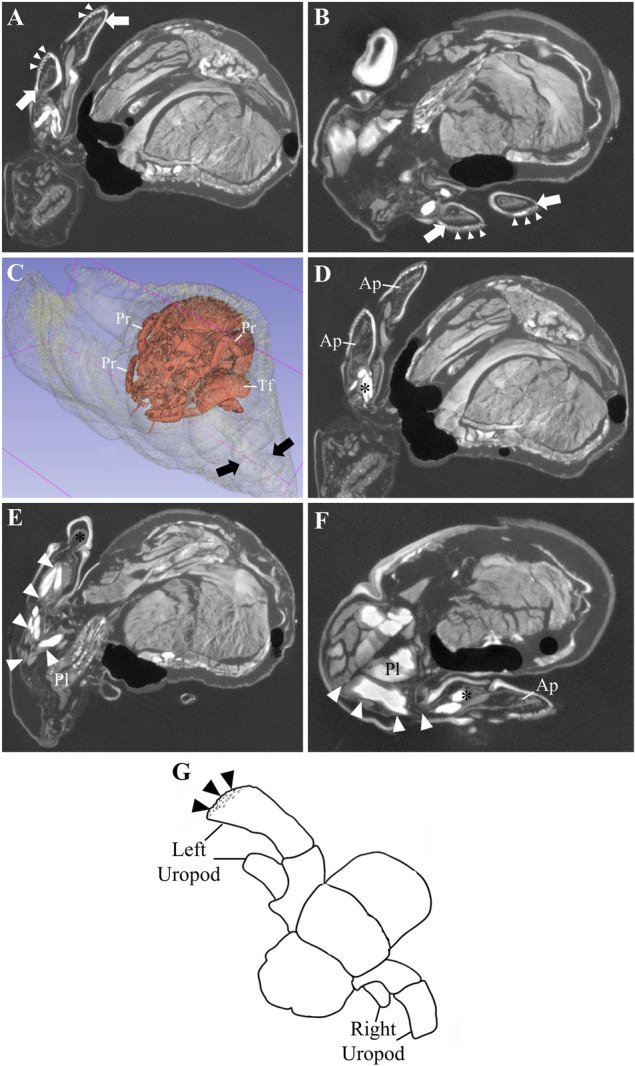
Internal morphology of *Calcinus tibicen* (Herbst, 1791). (A, B) General view of the micro‐computed tomography (*µCT*) images revealing the tight positioning of the uropods (white arrows) against the shell, reinforcing the idea that the scale setae (white arrowheads) serve to enhance the grip of the hermit crab to the shell. (C) 3D reconstruction from the *µCT* data showing that the pleon of the hermit crab is situated in the region of the middle chamber instead of the innermost chamber (black arrows). (D, E) Connection between the uropod apodemes (on the left side), coxopodite (black asterisk), and the musculature extending from the pleon (white arrowheads). (F) Connection between the uropod apodemes (on the right side), coxopodite (black asterisk), and the musculature extending from the pleon (white arrowheads). (G) A scheme showing the position at which the tailfan was scanned. Scheme adapted from Harvey (1998). A similarity in the anatomical arrangement between the left and right sides of the body is observed, although the muscle fibers connected to the left uropod appear to be larger than those connected to the right uropod. Black arrowheads indicate the scale setae. Ap, Apodeme; Pl, Pleon; Pr, Pereopods; Tf, Tailfan.

The *µCT* images revealed that the uropods are positioned tightly against the shell, indicating that the scale setae observed earlier function as anchoring structures to enhance the grip of hermit crabs within their shells (Figure 4A,B). Additionally, it was observed that the hermit crab occupies the middle chambers of the shells rather than the innermost ones (Figure 4C). Within the left uropod, there is a delicate cuticle structure known as apodeme (Figure 4D), which connects to the musculature on the coxopodite of the uropod and further extends to the pleon musculature (Figure 4E). The same anatomical arrangement is observed in the right uropod (Figure 4F), although the muscle fibers on this side appear smaller compared to those in the left uropod.

### 3.3 Histology Characterization of Tailfan's Tissues

**Figure 5:**
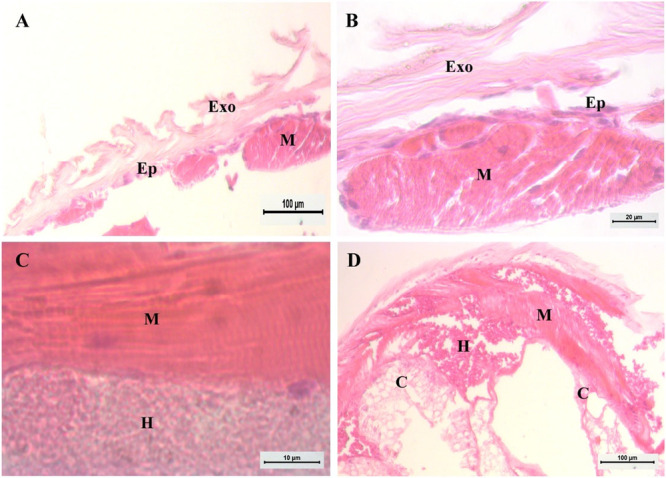
Tailfan of *Calcinus tibicen* (Herbst, 1791) by histology. (A) A detail of the telson demonstrates that the exoskeleton consists of a thick layer of chitinous cuticle on the rasping surface. (B) The presence of epithelial cells in the layer below the exoskeleton. (C) Striated muscle tissue with apparent myocytes arranged in the same longitudinal orientation. (D) The core shows connective tissue and the hemolymphatic space. The scale bar in A and D corresponds to 100 µm, while the scale bar in B and C corresponds to 20 µm and 10 µm, respectively. C – connective tissue; Ep, Epthelial cells layer; Exo, Exoskeleton; H, hemolymph; M, myocytes.

The histology of the distal part of the pleon in transition to the shell anchoring revealed a complex crustacean appendage comprised of several specialized tissue types, often described as the terminal anchoring system of the soft pleon (Figure 5). The exoskeleton is a thick layer of chitinous cuticle that is highly adapted to form a specialized rasping surface (Figure 5A). Located below this layer, there is another layer of epithelial cells (Figure 5B). The required force for gripping and retraction is provided by myocytes from striated muscle tissue (Figure 5C). Supporting these structures is the core, which contains loose connective tissue and the hemolymphatic space, serving as the circulatory pathway for hemolymph (Figure 5D).

## 4 Discussion

The anatomical functionality of hermit crabs has received limited attention in recent years. However, previous studies from the 60's and 70's (Chapple 1966b, 1969c, 1969b, 1973; Bent and Chapple 1977) and even older (Thorson 1946; Elmhirst 1947; Bott 1949; Brightwell 1951) have provided insights into how hermit crabs grip their shells using the available resources at the time. The present study has addressed some previously unanswered questions, shedding light on this aspect of hermit crab biology.

The fourth and fifth pair of pereopods primarily exhibit scale setae, which are likely the “rasping structures” mentioned in Bott (1949). These setae are overlapping structures with space between them. They are located on the external surface of the pereopods and, when inside the shells, these setae interacts with the rough inner shell surface. Additionally, the dactyl of the fourth pair of pereopods has overlapping teeth on its external surface, which also makes direct contact with the shell. The broader role of the fourth and fifth pairs of pereopods as a supportive mechanism for shell support has been described previously (Thorson 1946; Elmhirst 1947; Bott 1949; Brightwell 1951). However, these studies used a rustic glass shell and described the role of the fourth and fifth pairs of pereopods based on their anatomical positions. Brightwell (1951) provides a detailed description of the glass shells employed. The present study has demonstrated, from a finer‐scale perspective, that these pereopods function as support structures aiding the hermit crab to have better stability and grip when holding the shell. The scale setae on both pereopods, along with the teeth on the external surface of the dactyl of the fourth pair of pereopods, scrape onto the inner rough shell surface and locks into it, thus providing a firm grip. It is also important to highlight the function of P4 and P5 as grooming structures since it has setae with shape that enables rasping movements (Keiler and Richter 2011).

The potential role of the tailfan, especially the uropods, in anchoring the shell has been hypothesized in previous literature (Elmhirst 1947; Bott 1949; Brightwell 1951; Lane and Briffa 2020). The findings of the present study indicate that the telson is primarily smooth with a sparse distribution of simple setae, suggesting it does not play a significant role in supporting the shell. However, the uropods (both right and left) do indeed function as a hooking mechanism. The exopod and endopod of both uropods are oriented at different planes. The three‐dimensional reconstructions (via *µCT*) revealed that when inside the shells, the tailfan is curled against shells' columella, allowing the outer surface of the uropods to interact with the inner surface of the shells. The presence of overlapping scale setae on the outer surface of both uropods serves as a hooking structure when the hermit crabs press them against the rough surface of the inner shells. The plumose setae along the margin of the uropods may serve as cleaning structures, potentially removing or obstructing any fine particles on the shell. Consequently, when the hermit crabs press their uropods, along with their scale setae, against the shell's surface, it creates a firm grip, effectively hooking the animal to the shell.

The functionality of the uropods is mediated by their musculature. Whitin the endopod and exopod, there is a fine cuticle called apodeme, which is connected to the musculature located in the coxopodite of the uropod. This musculature is attached to the axial body in the pleonal region. To facilitate movement and enable the attachment of the scale setae inside the shell, providing a stronger grip, the muscles in the coxopodite contract, transferring the generated energy to the apodeme. This mechanism is similar to the closing of the claw, where the muscles in the propodus transfer energy to the apodeme inside the dactyl, causing the movable finger to close (Palaoro et al. 2020; Levinton and Weissburg 2021).

When examining the position of hermit crabs inside the shell, it becomes evident that the tailfan is aligned with the fourth and fifth pereopods. This alignment suggests that these structures function as both support and hooks, creating multiple anchor points within the same plane. This orientation allows the hermit crabs to firmly grip their shells during movement and combat. Observations of hermit crab fights by Lane and Briffa (2020) revealed that these organisms specifically targeted certain regions of the shell to dislodge their opponents. The authors noted that these attacks were focused on the anchor points, aiming to remove the defender and claim the shell. Our findings, in conjunction with those of Lane and Briffa (2020), indicate that these attacked regions correspond to the plane where the fourth and fifth pereopods and uropods are positioned. Therefore, this region can be considered an anchor region due to the presence of anchoring structures in the uropods, fourth, and fifth pereopods that press against the inner surface of the shell.

The functional morphology of the uropod, as suggested by the external features and the internal musculature, is fundamentally supported by its histological composition. The µCT analysis revealed a significant muscle presence within the uropods, particularly the left one, indicating a high demand for actuation. Complementing this, histological results showed that the musculature present in the tailfan region is of the striated type, which is essential for providing the force and fine motor control necessary for sustained anchoring (William D Chapple 1969). This striated muscle tissue is robustly attached to the inner cuticular surface in the basal segments, confirming a powerful mechanical system. This muscular arrangement, combined with the epithelial layer responsible for maintaining the cuticular integrity, enables the crab to move and manipulate the uropods, allowing the external structures (e.g., scale setae) to firmly engage with the internal granularity of the shell's central column.

The present study provides new insights into the anatomical functionality of hermit crabs, emphasizing the crucial role of the pereopods and tailfan in stabilization and fixation within shells. Building on previous studies and utilizing modern three‐dimensional reconstruction techniques, it has been demonstrated that hermit crabs use scale setae on their pereopods and uropods as anchoring mechanisms, ensuring a firm grip on the inner surface of the shells. This investigation not only elucidates the importance of these appendages in the biology of hermit crabs but also offers a solid foundation for future studies on the behavioral and evolutionary ecology of these organisms.

## Author Contributions

**Alexandre R. da Silva:** conceptualization, data curation, formal analysis, funding acquisition, investigation, methodology, project administration, software, validation, visualization, writing – original draft, writing – review and editing. **Carolina Siqueira Safra Terra Melo:** anatomical and histological processing assay, methodology, writing – review. **Giselle Pinto de Faria Lopes:** histological analysis, funding, writing – review and editing. **Caio S. Nogueira:** formal analysis, investigation, writing – original draft, writing – review and editing.

## Conflicts of Interest

The authors declare no conflicts of interest.

## Data Availability

The data that support the findings of this study are available on request from the corresponding author. The data are not publicly available due to privacy or ethical restrictions.
